# Coenzyme Q_10_ Supplementation in Aging and Disease

**DOI:** 10.3389/fphys.2018.00044

**Published:** 2018-02-05

**Authors:** Juan D. Hernández-Camacho, Michel Bernier, Guillermo López-Lluch, Plácido Navas

**Affiliations:** ^1^Centro Andaluz de Biología del Desarrollo and CIBERER, Instituto de Salud Carlos III, Universidad Pablo de Olavide-CSIC-JA, Sevilla, Spain; ^2^Translational Gerontology Branch, National Institute on Aging, National Institutes of Health, Baltimore, MD, United States

**Keywords:** Coenzyme Q, aging, disease, mitochondria, antioxidant, CoQ deficiency

## Abstract

Coenzyme Q (CoQ) is an essential component of the mitochondrial electron transport chain and an antioxidant in plasma membranes and lipoproteins. It is endogenously produced in all cells by a highly regulated pathway that involves a mitochondrial multiprotein complex. Defects in either the structural and/or regulatory components of CoQ complex or in non-CoQ biosynthetic mitochondrial proteins can result in a decrease in CoQ concentration and/or an increase in oxidative stress. Besides CoQ_10_ deficiency syndrome and aging, there are chronic diseases in which lower levels of CoQ_10_ are detected in tissues and organs providing the hypothesis that CoQ_10_ supplementation could alleviate aging symptoms and/or retard the onset of these diseases. Here, we review the current knowledge of CoQ_10_ biosynthesis and primary CoQ_10_ deficiency syndrome, and have collected published results from clinical trials based on CoQ_10_ supplementation. There is evidence that supplementation positively affects mitochondrial deficiency syndrome and the symptoms of aging based mainly on improvements in bioenergetics. Cardiovascular disease and inflammation are alleviated by the antioxidant effect of CoQ_10_. There is a need for further studies and clinical trials involving a greater number of participants undergoing longer treatments in order to assess the benefits of CoQ_10_ treatment in metabolic syndrome and diabetes, neurodegenerative disorders, kidney diseases, and human fertility.

## Introduction

Coenzyme Q (CoQ, ubiquinone) is a unique lipid-soluble antioxidant that is produced *de novo* in animals (Laredj et al., 2014). It is composed of a benzoquinone ring and a polyisoprenoid tail containing between 6 and 10 subunits that are species-specific and confers stability to the molecule inside the phospholipid bilayer. The isoprene chain in *Saccharomyces cerevisiae* contains six subunits (CoQ_6_), seven subunits are present in *Crucianella maritima* (CoQ_7_), eight in *E. coli* (CoQ_8_), nine and 10 in mice (CoQ_9_ and CoQ_10_), and 10 in humans (CoQ_10_).

**Figure 1:**
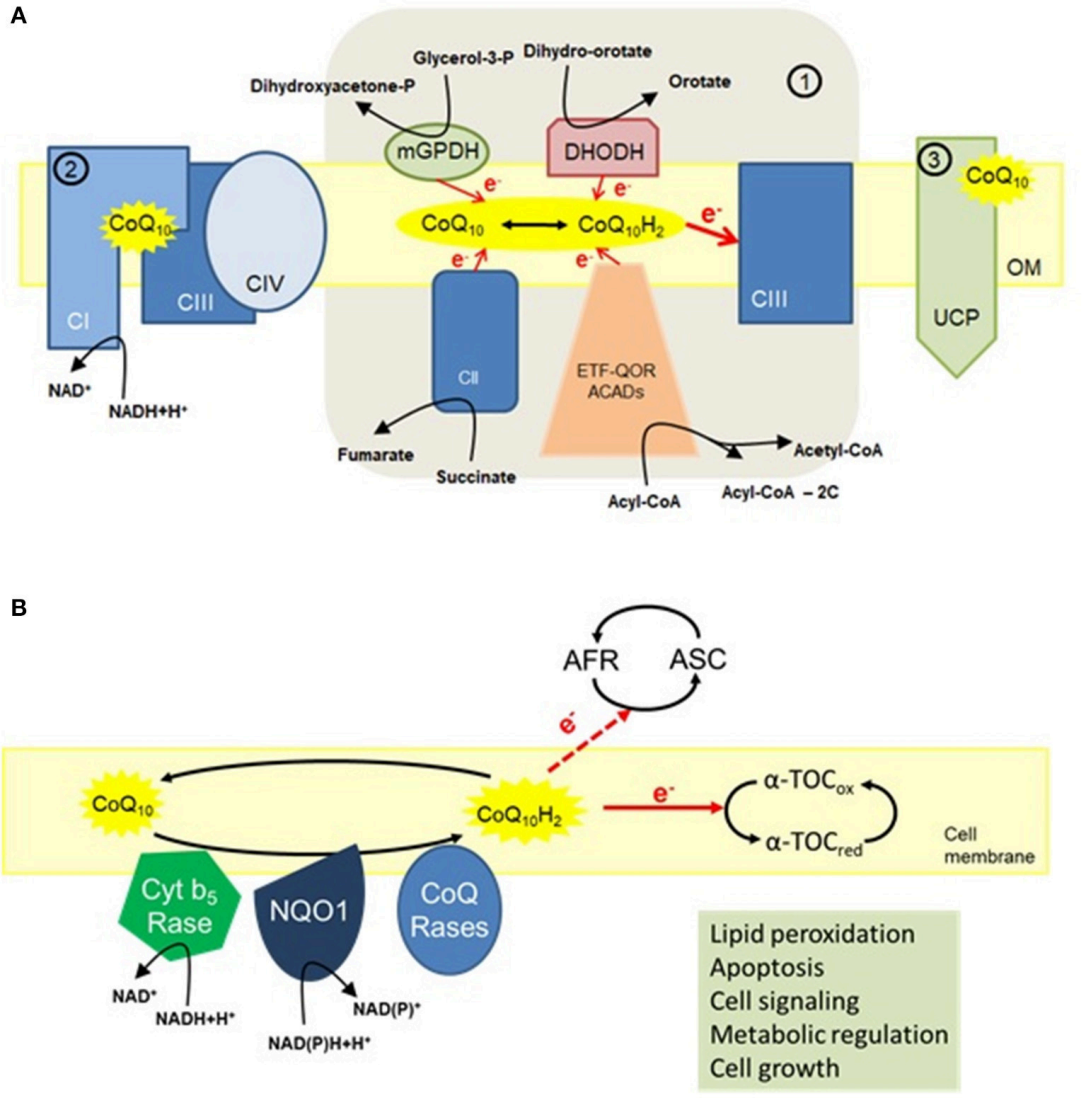
The multiple functions of CoQ_10_. **(A)** Mitochondria. (1) The main function of CoQ_10_ in mitochondria is to transfer electrons to complex III (CIII). By transferring two electrons to CIII, the reduced form of CoQ_10_ (ubiquinol) is oxidized to ubiquinone. The pool of ubiquinol can be restored by accepting electrons either from members of the electron transport chain (CI and CII), glycerol-3-phosphate dehydrogenase (GPDH) and dihydroorotate dehydrogenase (DHODH) that use cytosolic electron donors, or from acyl-CoA dehydrogenases (ACADs); (2) CoQ_10_ is also a structural component of both CI and CIII and is associated with respiratory supercomplexes, especially the depicted supercomplex I+III+IV; (3) CoQ_10_ is an obligatory factor in proton transport by uncoupling proteins (UCPs) with concomitant regulation of mitochondrial activity (López-Lluch et al., 2010). **(B)** Cell membrane activities of CoQ_10_. Present in nearly all cellular membranes, CoQ_10_ offers antioxidant protection, in part, by maintaining the reduced state of α-tocopherol (α-TOC) and ascorbic acid (ASC). Furthermore, CoQ_10_ also regulates apoptosis by preventing lipid peroxidation. Other functions of CoQ_10_ in cell membrane include metabolic regulation, cell signaling, and cell growth through local regulation of cytosolic redox intermediates such as NAD(P)H (López-Lluch et al., 2010).

CoQ is a central component in the mitochondrial electron transport chain (ETC) located in the inner mitochondrial membrane where it transports electrons from complexes I and II to complex III to provide energy for proton translocation to the intermembrane space (López-Lluch et al., 2010). CoQ is also a structural component in complexes I and III and is essential in the stabilization of complex III in yeast (Santos-Ocana et al., 2002; Tocilescu et al., 2010). The ETC complexes are assembled into respiratory supercomplexes in order to function efficiently and prevent electron leakage to oxygen that ultimately results in the production of reactive oxygen species (ROS) (Genova and Lenaz, 2014; Guo et al., 2017; Milenkovic et al., 2017). Mitochondrial CoQ may be associated in discrete pools dedicated to either NADH-coupled or FADH_2_-coupled electron transport (Lapuente-Brun et al., 2013). Complex I stability is determined by CoQ redox state (Guaras et al., 2016) and the reduced form of CoQ (CoQH_2_) directs complex I-specific ROS production to extend lifespan in *Drosophila* (Scialo et al., 2016). Mitochondrial activities such as the dihydroorotate dehydrogenase, β-oxidation of fatty acids, and mitochondrial glycerol-3-phosphate dehydrogenase contribute also to the increase in CoQH_2_ levels (Alcazar-Fabra et al., 2016) (Figure 1A).

CoQ provides antioxidant protection to cell membranes and plasma lipoproteins (López-Lluch et al., 2010). By lowering lipid peroxidation of low-density lipoprotein **(**LDL) particles that contributes to atherosclerosis (Thomas et al., 1997), CoQ treatment confers health benefits against cardiovascular diseases (Mortensen et al., 2014; Alehagen et al., 2016). The anti-oxidant function of CoQ is especially important in the plasma membrane by reducing vitamins C and E, and in preventing ceramide-mediated apoptosis (Navas et al., 2007), an important regulator of lifespan in the context of normal aging (De Cabo et al., 2004; López-Lluch et al., 2005; Martin-Montalvo et al., 2016) (Figure 1B). It has been proposed that NAD(P)H:quinone oxidoreductase 1 (NQO1) acts as a redox-sensitive switch to regulate the response of cells to changes in the redox environment (Ross and Siegel, 2017). The pharmacokinetics variability of the different compositions of CoQ_10_ may result in fairly different plasma concentration-time profiles after CoQ_10_ administration (Weis et al., 1994; Molyneux et al., 2004). Indeed, the major amount of orally supplemented CoQ_10_ is eliminated via feces, with only a fraction of ingested CoQ_10_ reaching the blood and ultimately the various tissues and organs (Bentinger et al., 2003).

For these reasons, CoQ appears suitable for use in the treatment of different diseases. Here, we present recent advances in CoQ_10_ treatment of human diseases and the slowing down of the aging process, and highlight new strategies aimed at delaying the progression of chronic diseases by CoQ_10_ supplementation.

## CoQ_10_ biosynthesis pathway

CoQ_10_ biosynthesis pathway is initiated in the cytosol where the isoprene tail is made from the conversion of mevalonate, a key intermediate involved in the synthesis of cholesterol and dolichol and protein prenylation adducts (Trevisson et al., 2011). The end of the isoprene tail is formed by a cytosolic heterotetrameric protein complex encoded by *PDSS1* and *PDSS2* genes (*COQ1*) (Kawamukai, 2015). The quinone ring unit is also produced in the cytosol from tyrosine or phenylalanine and attached to the isoprene tail inside mitochondria through the activity of *COQ2*-encoded polyprenyl transferase (Laredj et al., 2014; Acosta et al., 2016). The benzoquinone ring is then modified in the inner mitochondrial membrane and this process involves at least 12 nuclear-encoded proteins (*COQ*) (Bentinger et al., 2010), which are required for the formation of a multiprotein complex known as “synthome” (He et al., 2014; Alcazar-Fabra et al., 2016; Floyd et al., 2016). The assembly and stabilization of the synthome is far from being understood as it may encompass yet to be discovered new interacting protein partners (Allan et al., 2015; Morgenstern et al., 2017). CoQ biosynthesis pathway is tightly regulated both at the transcriptional and translational levels (Turunen et al., 2000; Brea-Calvo et al., 2009; Cascajo et al., 2016) and by phosphorylation of some of the complex components (Martin-Montalvo et al., 2011, 2013; Guo et al., 2017; He et al., 2017).

## CoQ_10_ deficiency syndrome

CoQ_10_ deficiencies are based on decreased CoQ_10_ levels and can be measured in skeletal muscle and/or skin fibroblast from patients suffering these rare (frequency less than 1:100000) inherited clinically and genetically heterogeneous diseases that impair oxidative phosphorylation and other mitochondrial functions (Desbats et al., 2015b; Acosta et al., 2016; Gorman et al., 2016; Rodriguez-Aguilera et al., 2017). CoQ_10_ deficiency can be caused by mutations in *COQ* genes that encode proteins of the CoQ biosynthesis pathway (primary deficiency) or as a secondary deficiency caused by defects in other mitochondrial functions that are indirectly involved in the biosynthesis of CoQ_10_ (Doimo et al., 2014; Desbats et al., 2015a; Gorman et al., 2016; Yubero et al., 2016; Salviati et al., 2017).

Primary CoQ_10_ deficiency is characterized by highly heterogeneous clinical signs, with the severity and symptoms varying greatly as is the age of onset, which can be from birth to the seventh decade, and beyond (Salviati et al., 2017). Current clinical manifestations that may indicate primary CoQ_10_ deficiency are: (1) steroid-resistant nephrotic syndrome without mutations in *NPHS1* and/or *NPHS2* genes particularly when associated with deafness, retinopathy, and other neurological defects; (2) mitochondrial encephalopathy including hypotonia, strokes, cerebellar ataxia, spasticity, peripheral neuropathy, and intellectual disability. These patients may also be presenting symptoms of myopathy, retinopathy, optic atrophy, sensorineural hearing loss, and hypertrophic cardiomyopathy; (3) unexplained ataxia particularly when family history suggests a recessive autosomal heritage; and (4) exercise intolerance appearing from 6 to 33 years of age, with muscular weakness and high serum creatine kinase.

Primary CoQ_10_ deficiencies are conditions where pathogenic mutations have occurred in genes involved in the biosynthesis of CoQ_10_ (Table 1). Mutations in *PDSS2, COQ6*, and *ADCK4/COQ8B* affect mainly the kidney by inducing steroid-resistant nephrotic syndrome while *COQ2* mutations induce multisystem disorders whose severity correlates with the mutated genotype (Desbats et al., 2016). Individuals affected by pathogenic mutations in the deduced amino acid sequence of *COQ4, COQ7, COQ9*, and/or *PDSS1* develop encephalopathy and those affected by mutations in *ADCK3/COQ8A* develop mainly cerebellar disorders.

**Table 1:** Clinical phenotypes caused by mutations in CoQ synthome and the effect of CoQ_10_ therapy in humans.

**Gene**	**N° of patients**	**Age of onset**	**Clinical phenotype**	**Effect of CoQ therapy**	**References**
*PDSS1 (COQ1)*	2	1−2 years	Encephalopathy, Peripheral neuropathy, Optic atrophy, Heart valvulopathy, Mild lactic acidosis, Overweight, Deafness, Moderate pulmonary artery hypertension, Mild mental retardation	No	Laredj et al., 2014; Desbats et al., 2015a; Salviati et al., 2017
*PDSS2 (COQ1)*	4	~3 months	Nephrotic syndrome, Leigh syndrome, Ataxia, Deafness, Retinopathy	Improvement	Laredj et al., 2014; Desbats et al., 2015a,b; Salviati et al., 2017
*COQ2*	17	Birth, 3 weeks,~1 year, 18 month, Adolescence	Nephrotic syndrome, Encephalomyopathy, Hypertrophic cardiomyopathy, MELAS-like syndrome, Seizures, Retinopathy, Lactic acidosis, Deafness, Adult onset multisystem atrophy, Cerebellar atrophy, Myoclonus, Optic atrophy, Myopathy edema	Improvement	Jakobs et al., 2013; Laredj et al., 2014; Desbats et al., 2015a,b; Gigante et al., 2017
*COQ4*	1	Birth	Encephalomyopathy, Weakness, Hypotonia, Intellectual disability, Seizures, Heart failure, Myopathy, Hypertrophic cardiomyopathy, Myopathy, Dysmorphic features	Improvement	Salviati et al., 2012, 2017; Laredj et al., 2014; Desbats et al., 2015a; Sondheimer et al., 2017
*COQ6*	13	0.2–6 years	Nephrotic syndrome, Deafness, Encephalopathy, Seizures, Ataxia, Growth retardation, Facial dysmorphism	Improvement	Heeringa et al., 2011; Laredj et al., 2014; Desbats et al., 2015a; Salviati et al., 2017
*COQ7*	1	Birth	Encephalopathy, Intellectual disability, Peripheral neuropathy, Muscle weakness	Improvement	Freyer et al., 2015; Salviati et al., 2017
*ADCK3 (COQ8A)*	23	18 months, 1–2, 3–11, 15–18, 27 years	Cerebellar ataxia, Encephalopathy, Seizures, Dystonia, Spasticity, Migraine, Exercise intolerance, Myoclonus, Intellectual disability, Hypotonia, Muscle fragility, Feeding difficulties, Walking difficulty	Improvement	Laredj et al., 2014; Desbats et al., 2015a; Barca et al., 2016; Salviati et al., 2017
*ADCK4 (COQ8B)*	15	<1, 3–14, 16–21 years	Mental retardation, Nephrotic syndrome	Improvement	Ashraf et al., 2013; Laredj et al., 2014; Desbats et al., 2015a; Salviati et al., 2017
*COQ9*	1	Birth	Encephalomyopathy, Renal tubulopathy, Cardiac hypertrophy, Seizures, Cerebellar atrophy, Myopathy	No	Laredj et al., 2014; Desbats et al., 2015a; Salviati et al., 2017

Abnormally low CoQ_10_ levels can be associated with mitochondrial pathologies caused by mutations in genes encoding components of the oxidative phosphorylation chain or of other cellular functions not directly associated with mitochondrial function (Yubero et al., 2016). Known as secondary CoQ_10_ deficiencies, these disorders could represent an adaptive mechanism to bioenergetic requirements. For example, secondary CoQ_10_ deficiency can appear in some patients with defects in glucose transport caused by *GLUT1* mutations (Yubero et al., 2014). A group of patients with very severe neuropathies showed impaired CoQ_10_ synthesis, indicating the importance of CoQ_10_ homeostasis in human health (Asencio et al., 2016).

In individuals with primary CoQ_10_ deficiency, early treatment with high-dose oral CoQ_10_ supplementation improves the pathological phenotype, limits the progression of encephalopathy, and helps recover kidney damage (Montini et al., 2008). Onset of renal symptoms in *PDSS2*-deficient mice can be prevented with CoQ_10_ supplementation (Saiki et al., 2008). The European Medicine Agency (EMA) has recently approved ubiquinol—the reduced form of CoQ_10_ (CoQ_10_H_2_)—as an orphan drug for the treatment of primary CoQ_10_ deficiency (http://ec.europa.eu/health/documents/community-register/html/o1765.htm). However, patients suffering from secondary CoQ_10_ deficiency may fail to respond to CoQ_10_ supplementation (Pineda et al., 2010).

## CoQ_10_ and aging

A significant reduction in the rate of CoQ biosynthesis has been proposed to occur during the aging process and aging-associated diseases (Beyer et al., 1985; Kalen et al., 1989; Battino et al., 1995; Turunen et al., 1999). However, there are discrepancies about the relationship between the levels of CoQ and the progression of aging.

Mice lacking one of the alleles of the *COQ7* gene (*mCOQ7/mCLK1 gene*) show extended longevity even though their CoQ levels are the same as wild-type mice, suggesting that a factor other than CoQ *per se* may be responsible for lifespan extension in these animals (Lapointe and Hekimi, 2008). However, other *in vivo* studies have reported a direct association between longevity and mitochondrial levels of CoQ in the *Samp1* model of senescence-accelerated mice (Tian et al., 2014). Supplementation with ubiquinol has been shown to activate mechanisms controlling mitochondrial biogenesis (Schmelzer et al., 2010) and delay senescence (Tian et al., 2014).

The concentrations of CoQ_10_ in the plasma of elderly people are positively correlated with levels of physical activity and cholesterol concentrations (Del Pozo-Cruz et al., 2014a,b), as well as with lower lipid oxidative damage. The antioxidant protection conferred by CoQ_10_ is associated with skeletal muscle performance during aging as evidenced by the fact that a high CoQ_10_H_2_/CoQ_10_ ratio is accompanied by an increase in muscle strength (Fischer et al., 2016). Conversely, a low CoQ_10_H_2_/CoQ_10_ ratio could be predictor of sarcopenia in humans. Older individuals given a combination of selenium and CoQ_10_ over a 4-year period reported an improvement in vitality, physical performance, and quality of life (Johansson et al., 2015). Furthermore, CoQ_10_ supplementation confers health benefits in elderly people by preventing chronic oxidative stress associated with cardiovascular and neurodegenerative diseases (Gonzalez-Guardia et al., 2015). Despite these evidences, more reliable clinical trials focusing on the elderly are needed before considering CoQ_10_ as an effective anti-aging therapy (Varela-Lopez et al., 2016).

## CoQ_10_ supplementation in the treatment of human diseases

CoQ_10_ has been used in the treatment of a number of human pathologies and disorders. Clinical trials, systematic reviews, and meta-analyses have examined the safety and efficacy of CoQ_10_ in treating human diseases. With regards to safety, the highest dose for CoQ_10_ supplementation is 1200 mg daily according to well-designed randomized, controlled human trials, although doses as high as 3000 mg/day have been used in shorter clinical trials (Hathcock and Shao, 2006). CoQ is generally safe and well-tolerated in treating patients suffering from early-stage Huntington disease with 2400 mg/day of CoQ_10_ (McGarry et al., 2017).

As indicated below, prudence is needed when interpreting the results of several clinical trials. A combination of factors including the small number of trials, substantial differences that exist in the experimental designs, dose and duration of treatment, the number of patients enrolled, and the relative short follow-up periods contribute to apparent inconsistencies in the published data. Despite these limitations, CoQ_10_ can be considered as an important coadjuvant in the treatment of different diseases, especially in chronic conditions affecting the elderly.

### Cardiovascular disease

The number of deaths attributed to heart failure is increasing worldwide and has become a global health issue. Heart failure is accompanied by increased ROS formation, which can be attenuated with antioxidants. A systematic review has recently examined the efficacy of CoQ_10_ supplementation in the prevention of cardiovascular disease (CVD) without lifestyle intervention (Flowers et al., 2014). These authors interpreted the results to indicate a significant reduction in systolic blood pressure without improvements in other CVD risk factors, such as diastolic blood pressure, total cholesterol, LDL- and high-density lipoprotein (HDL)-cholesterol, and triglycerides. A second meta-analysis explored the impact of CoQ_10_ in the prevention of complications in patients undergoing cardiac surgery, and the results showed that CoQ_10_ therapy lowers the need of inotropic drugs and reduces the appearance of ventricular arrhythmias after surgery (de Frutos et al., 2015).

Short-term daily treatment (12 weeks or less) with 100 mg CoQ_10_ improves left ventricular ejection fraction in patients suffering from heart failure (Fotino et al., 2013). In contrast, no effect of CoQ_10_ was observed on left ventricular ejection fraction or exercise capacity in patients with heart failure (Madmani et al., 2014). However, a 2-year treatment with CoQ_10_ (300 mg/day) as adjunctive therapy in a randomized, controlled multicenter trial affecting 420 patients suffering from chronic heart failure (Q-SYMBIO trial) demonstrated an improvement in symptoms and reduction in major cardiovascular events (Mortensen et al., 2014). A study on the effects of long-term treatment with CoQ_10_ (200 mg/day) plus selenium (200 μg as selenized yeast) in a homogeneous Swedish healthy elderly population (*n* = 219) revealed a significant reduction in cardiovascular mortality not only during the 4-year treatment period, but also 10 years later, compared to those taking either a placebo (*n* = 222) or were without treatment (*n* = 227) (Alehagen et al., 2015, 2016).

### Metabolic syndrome and diabetes

CoQ_10_ has been proposed for the treatment of metabolic syndrome and type 2 diabetes by virtue of its antioxidant properties. However, current results from clinical trials cannot conclusively determine the efficacy of CoQ_10_ because either of the missing information on the CoQ_10_ formulation used or the low number of trials and/or patients enrolled.

One effect attributable to CoQ_10_ therapy in type 2 diabetic patients (260 mg/day for 11 weeks) is its rather mild, but significant capacity to reduce fasting plasma glucose levels without changes in fasting insulin and glycated hemoglobin (HbA1c) (Moradi et al., 2016). However, analysis of more than seven trials involving 356 participants showed that CoQ_10_ supplementation for at least 12 weeks had no significant effects on glycemic control, lipid profile, or blood pressure in diabetic patients, but was able to reduce serum triglycerides levels (Suksomboon et al., 2015). In a follow-up analysis of data obtained from Q-SYMBIO clinical trials (Mortensen et al., 2014), Alehagen and colleagues were able to show that in elderly healthy participants who received selenium and CoQ_10_ supplementation for over 4 years, an increase in insulin-like growth factor 1 (IGF-1) and postprandial insulin-like growth factor-binding protein 1 (IGFBP-1) levels, and greater age-corrected IGF-1 score based on the standard deviation of the mean value were observed compared with placebo-treated individuals (Alehagen et al., 2017).

Supplementation with CoQ_10_ has produced beneficial effects in the treatment of hypercholesterolemia and hypertriglyceridemia by initiating changes in blood lipid concentration. A combination of CoQ_10_ with red yeast rice, berberina, policosanol, astaxanthin, and folic acid significantly decreased total cholesterol, LDL-cholesterol, triglycerides, and glucose in the blood while increasing HDL-cholesterol levels (Pirro et al., 2016). However, the impact of CoQ_10_ alone without the other supplements was not directly assessed. Nevertheless, there are reports to suggest that CoQ_10_ is very effective in reducing serum triglycerides levels (Suksomboon et al., 2015) and plasma lipoprotein(a) (Sahebkar et al., 2016). Chronic treatment with statins is associated with myopathy (Law and Rudnicka, 2006), a side-effect representing a broad clinical spectrum of disorders largely associated with a decrease in CoQ_10_ levels and selenoprotein activity (Thompson et al., 2003; Fedacko et al., 2013). Statins impair skeletal muscle and myocardial bioenergetics (Littarru and Langsjoen, 2007) via inhibition of 3-hydroxy-3-methylglutaryl-CoA (HMG-CoA) reductase, a key enzyme in the mevalonate pathway implicated in cholesterol and CoQ biosynthesis, and reduction in mitochondrial complex III activity of the electron transport chain (Schirris et al., 2015). A total of 60 patients suffering from statin-associated myopathy were enrolled in a 3-month study to test for efficacy of CoQ_10_ and selenium treatment. A consistent reduction in their symptoms, including muscle pain, weakness, cramps, and fatigue was observed, suggesting an attenuation of the side-effects of chronic statin treatment following CoQ_10_ supplementation (Fedacko et al., 2013). In a previous study, however, 44 patients suffering from statin-induced myalgia saw no improvement in their conditions after receiving CoQ_10_ for 3 months (Young et al., 2007). Other studies have determined that CoQ_10_ supplementation improves endothelial dysfunction in type 2 diabetic patients treated with statins (Hamilton et al., 2009) and can reverse the worsening of the diastolic function induced by statins (Silver et al., 2004).

Because of its capacity to reduce the side-effects of statins, CoQ_10_ has been proposed to prevent and/or slow the progression of frailty and sarcopenia in the elderly chronically treated with statins.

### Kidney disease

Oxidative stress plays an essential role in diabetic kidney disease, and experiments performed on rats showed a promising protective effect of ubiquinol in the kidneys (Ishikawa et al., 2011). However, a meta-analysis study examining the efficiency of antioxidants on the initiation and progression of diabetic kidney disease revealed that antioxidants, including CoQ_10_, did not have reliable effects against this disease (Bolignano et al., 2017). Yet, in a recent clinical trial with 65 patients undergoing hemodialysis, supplementation with high amounts of CoQ_10_ (1200 mg/day) lowered F2-isoprostane plasma levels indicative of a reduction in oxidative stress (Rivara et al., 2017).

### Inflammation

Chronic inflammation and oxidative stress are associated with many age-related diseases such as cardiovascular diseases, diabetes, cancer, and chronic kidney disease. A recent meta-analysis explored the efficacy of CoQ_10_ on the plasma levels of C-reactive protein, interleukin 6 (IL-6) and tumor necrosis factor alpha (TNF-α) in patients afflicted with pathologies in which inflammation was a common factor including cardio-cerebral vascular disease, multiple sclerosis, obesity, renal failure, rheumatoid arthritis, diabetes, and fatty liver disease (Fan et al., 2017). Administration of CoQ_10_ in doses ranging from 60 to 500 mg/day for a 1-week to 4-month intervention period significantly decreased production of inflammatory cytokines. The authors also surmised that CoQ_10_ supplementation decreased pro-inflammatory cytokines and inflammatory markers in the elderly with low CoQ_10_ levels (Fan et al., 2017).

Metabolic diseases, characterized by chronic, low grade inflammation, respond well to CoQ_10_ supplementation with significant decrease in TNF-α plasma levels without having an effect on C-reactive protein and IL-6 production (Zhai et al., 2017). Rheumatoid arthritis patients receiving CoQ_10_ (100 mg/day) for 2 months tended to have lower TFN-α plasma levels than placebo-treated patients (Abdollahzad et al., 2015). Another study reported that CoQ_10_ therapy in doses ranging from 60 to 300 mg/day caused no significant decrease in C-reactive protein while eliciting a significant reduction in IL-6 levels (Mazidi et al., 2017). More recently, CoQ_10_ has been found to markedly attenuate the elevated expression of inflammatory and thrombotic risk markers in monocytes of patients with antiphospholipid syndrome, thereby improving endothelial function and mitochondrial activity in these patients (Perez-Sanchez et al., 2017).

A proinflammatory profile has also been associated with the progression of neurological symptoms in Down syndrome patients (Wilcock and Griffin, 2013). These patients have low CoQ_10_ plasma levels together with high plasma levels of proinflammatory cytokines, such as IL-6 and TNF-α (Zaki et al., 2017). Supplementation with CoQ_10_ confers protection against the progression of oxidative damage and mitochondrial dysfunction in Down syndrome patients (Tiano and Busciglio, 2011; Tiano et al., 2011).

### Neurodegenerative diseases

Mitochondrial dysfunction has been associated with the onset and/or development of neurodegenerative diseases (Arun et al., 2016; Bose and Beal, 2016; Grimm et al., 2016). Preclinical studies demonstrated that CoQ can preserve mitochondrial function and reduce the loss of dopaminergic neurons in the case of Parkinson's disease (Schulz and Beal, 1995). Experimental studies in animal models suggest that CoQ_10_ may protect against neuronal damage caused by ischemia, atherosclerosis, and toxic injury (Ishrat et al., 2006). Further, a screening for oxidative stress markers in patients with Parkinson's disease reported lower levels of CoQ_10_ and α-tocopherol and higher levels of lipoprotein oxidation in the plasma and cerebrospinal fluid compared to non-affected individuals (Buhmann et al., 2004). Moreover, CoQ_10_ deficiency was observed at a higher frequency in Parkinson's disease, underscoring its utility as a peripheral biomarker (Mischley et al., 2012). For this reason, it has been suggested that CoQ_10_ supplementation could benefit patients suffering from neurodegenerative diseases.

Studies in humans have shown that CoQ_10_ is safe and well-tolerated even at high doses (1200–2400 mg/day) although its effect on reversing functional decline of mitochondria is unclear (Schulz and Beal, 1995; McGarry et al., 2017). Two reviews on recent clinical trials testing CoQ_10_ supplementation reported the lack of improvement in motor functions in patients with neurodegenerative diseases, which led the authors to conclude that the use of CoQ_10_ in these patients is unnecessary (Liu and Wang, 2014; Negida et al., 2016). However, other clinical trials in patients suffering from Parkinson's, Huntington's, and Friedreich's ataxia suggest that CoQ_10_ supplementation could delay functional decline, particularly with regard to Parkinson's disease (Beal, 2002; Shults, 2003). Indeed, four randomized, double-blind, placebo-controlled studies comparing CoQ_10_ treatment in 452 patients at early or mid-stage Parkinson's disease reported improvements in daily activities and other parameters (Liu et al., 2011). In contrast, a more recent multicenter randomized, double-blind, and placebo-controlled trial with CoQ_10_ in 609 patients with early-stage Huntington's disease did not slow the rate of patients' functional decline (McGarry et al., 2017). There is not enough evidence to indicate that CoQ_10_ supplementation can delay the progression of Huntington's disease, at least in its early stages.

Initiated in 2006, the Alzheimer's Disease Cooperative Study evaluates the safety, tolerability, and impact of different antioxidants on biomarkers in this disease. There was no improvement observed in oxidative stress or neurodegeneration markers in a randomized clinical trial in Alzheimer's Disease patients with CoQ_10_ supplementation at a dose of 400 mg/day for 16 weeks (Galasko et al., 2012).

The role of plasma membrane CoQ_10_ in autism has been recently proposed (Crane et al., 2014). Patients with autistic spectrum disorders (ASDs) exhibit higher proportions of mitochondrial dysfunctions than the general population (Rossignol and Frye, 2012), as evidenced by developmental regression, seizures, and elevated serum levels of lactate or pyruvate in ASD patients. Treatment with carnitine, CoQ_10_, and B-vitamins confers some improvements in ASD patients (Rossignol and Frye, 2012; Gvozdjakova et al., 2014).

Alleviation of symptoms of chronic fatigue syndrome/myalgic encephalomyelitis has been reported after supplementation with a combination of NADH and CoQ_10_ (Campagnolo et al., 2017); however, these authors suggest that nutritional supplements in the mitigation of the symptoms of this disease are not currently justifiable.

### Human fertility

Male infertility has been associated with oxidative stress, and CoQ_10_ levels in seminal fluid is considered an important biomarker of healthy sperm (Gvozdjakova et al., 2015). Administration of CoQ_10_ improves semen parameters in the treatment of idiopathic male infertility (Arcaniolo et al., 2014). Additionally, CoQ_10_ supplementation (200–300 mg/day) in men with infertility improves sperm concentration, density, motility, and morphology (Safarinejad et al., 2012; Lafuente et al., 2013).

With regard to female infertility, the decrease in mitochondrial activity associated with CoQ_10_ deficiency probably affects the granulosa cells' capacity to generate ATP (Ben-Meir et al., 2015b). Indeed, reduction of CoQ_10_ levels in oocyte-specific *PDSS2*-deficient mice results in oocyte deficits and infertility (Ben-Meir et al., 2015a). Despite the absence of previous clinical trials that evaluate the effectiveness of CoQ_10_ supplementation in female infertility, these studies show promising results of this natural supplement in boosting female fertility during the prime reproductive period.

## Concluding remarks

**Figure 2:**
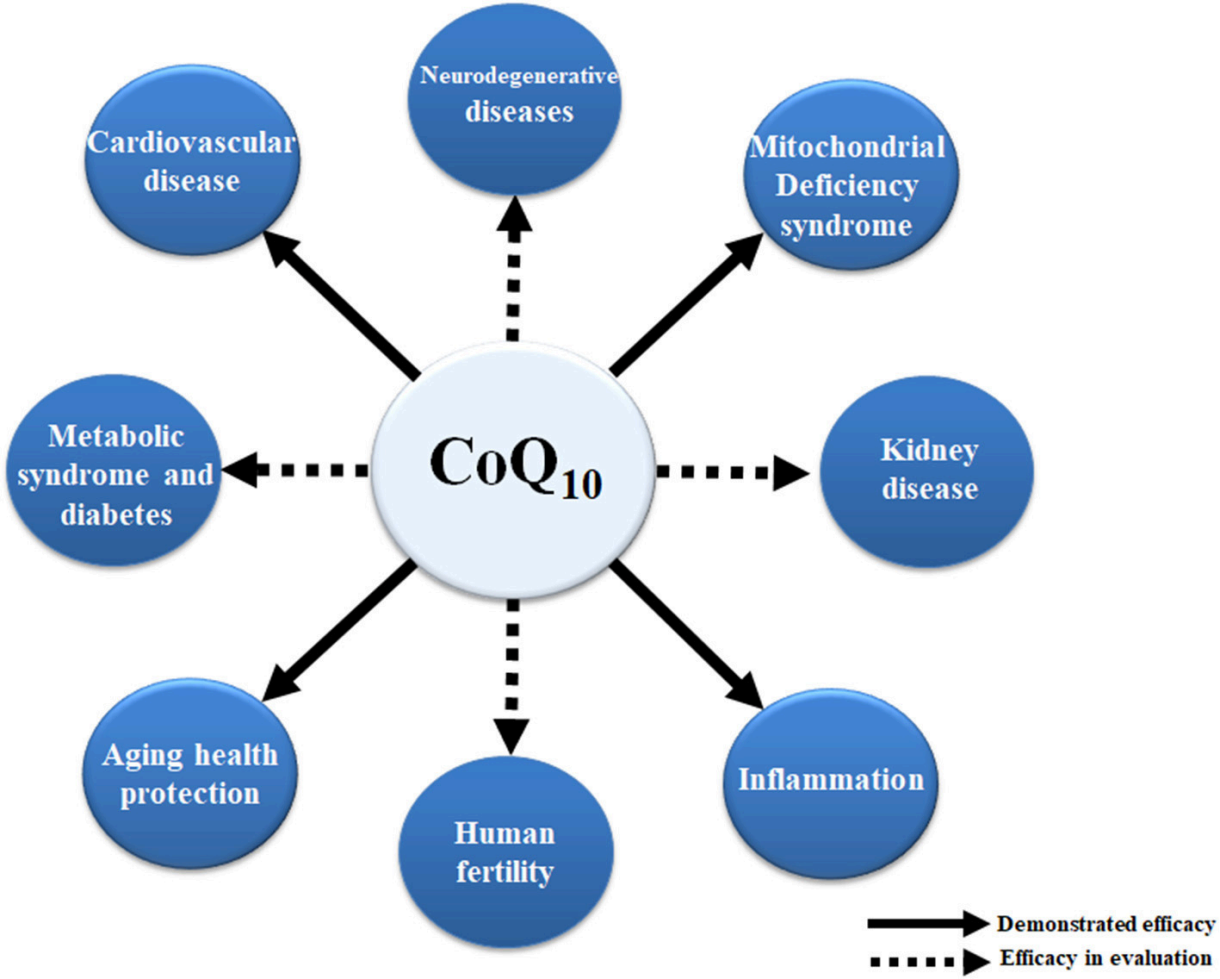
Effects of CoQ_10_ in human diseases. The positive effect of CoQ_10_ has been already demonstrated in mitochondrial syndromes associated with CoQ_10_ deficiency, inflammation, and cardiovascular diseases as well as in the delay of some age-related processes. Dashed lines depict other positive effects of CoQ_10_ with regard to kidney disease, fertility, metabolic syndrome, diabetes, and neurodegenerative diseases. However, more research is needed to validate these observations.

CoQ_10_ deficiency can be associated with a number of human diseases and age-related chronic conditions. In some cases, an unbalanced equilibrium between CoQ_10_ levels and/or functional ETC leads to mitochondrial dysfunction. In other cases, deficiency in CoQ_10_ and its associated antioxidative activity can significantly increase the level of oxidative damage. It seems clear that supplementation with CoQ_10_ improves mitochondrial function and confers antioxidant protection for organs and tissues affected by various pathophysiological conditions. The ability of CoQ_10_ to protect against the release of proinflammatory markers provides an attractive anti-inflammatory therapeutic for the treatment of some human diseases and in aging (Figure 2).

Following intraperitoneal administration of CoQ_10_ in rat, only small amount of the supplement reaches the kidney, muscle, and brain. Likewise, only a fraction of the orally administered CoQ_10_ reaches the blood while the major amount is eliminated via feces (Bentinger et al., 2003). The absoption of CoQ_10_ is slow and limited due to its hydrophobicity and large molecular weight and, therefore, high doses are needed to reach a number of rat tissues (e.g., muscle and brain) (Bhagavan and Chopra, 2006) and we can only assume that this also happens in humans. The pharmacokinetics variability of the different compositions of CoQ_10_ (Weis et al., 1994; Molyneux et al., 2004) may result in fairly different plasma concentration-time profiles after CoQ_10_ administration in the treatment of various diseases and monitoring of clinical effects.

Systematic reviews and meta-analyses have revealed that there are few randomized clinical trials on the effect of CoQ_10_ in combatting disease progression and improving quality of life. The results of these trials have been inconsistent likely due to varied dosages, small sample size, and short follow-up periods. More studies performed on humans in focused trials are needed in order to understand the promising effects of CoQ_10_.

## Author contributions

All authors listed have made a substantial, direct and intellectual contribution to the work, and approved it for publication.

### Conflict of interest statement

The authors declare that the research was conducted in the absence of any commercial or financial relationships that could be construed as a potential conflict of interest.
